# *In-vitro *Cellular Uptake and Transport Study of 9-Nitrocamptothecin PLGA Nanoparticles Across Caco-2 Cell Monolayer Model

**Published:** 2011

**Authors:** Katayoun Derakhshandeh, Gunther Hochhaus, Simin Dadashzadeh

**Affiliations:** a *Department of Pharmaceutics, School of Pharmacy, Kermanshah University of Medical Sciences, Kermanshah, Iran.*; b *Department of Pharmaceutics, School of Pharmacy, Florida, Gainesville, USA. University of Medical.*; c *Department of Pharmaceutics, School of Pharmacy, Shaheed Beheshti University of Medical Sciences, Tehran, Iran.*

**Keywords:** 9-Nitrocamptothecin, Nanoparticles, Uptake, Transport, Caco-2 cell

## Abstract

The uptake and transport of 9-nitrocamptothecin (9-NC), a potent anticancer agent, across Caco-2 cell monolayers was studied as a free and PLGA nanoparticle loaded drug.

Different sizes (110 to 950 nm) of 9-nitrocamptothecin nanoparticles using poly (lactic-glycolic acid) were prepared by via the nanoprecipitation method. The transport of nanoparticles across the Caco-2 cell monolayer as a function of incubation time and concentration was evaluated for each different nanoparticle formulation. The amount of 9-NC transported from the apical to the basolateral side and the uptake of the drug into the cells was determined by HPLC.

The uptake of intact nanoparticles into Caco-2 cells was visualized by confocal laser scanning microscopy using 6-coumarin as a fluorescent marker. The study demonstrated that Caco-2 cell uptake and transport of encapsulated 9-nitrocamptothecin is significantly affected by the diameter of the carrier and incubation time. In addition it was shown to be independent of concentration.

The results indicated a significant accumulation of the drug in the cell membrane and an enhanced diffusion across the cell membrane. There was also a sustained release of characteristics pertaining to polymeric carriers that provided prolonged drug availability for absorptive cells.

## Introduction

**Figure 1:**
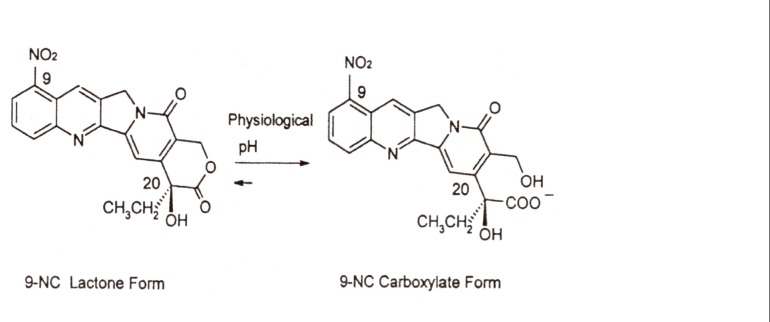
Chemical structures and equilibrium reaction between the lactone and carboxylate forms of 9-nitrocamptothecin (9-NC).

9-Nitrocamptothecin (9-NC, Figure 1) is a novel anticancer drug. It is an analogue of the natural plant alkaloid camptothecin that has shown high antitumor activity against advanced pancreatic carcinoma, ovarian epithelial cancer and leukemia and is currently in the end stage of clinical trials (1-4). The mode of action of camptothecins involves targeting the nuclear enzyme topoisomerase I. Unfortunately, these agents undergo at physiological pH rapid and reversible hydrolysis from a closed lactone form to an inactive hydroxy carboxylated one. This pH dependent hydrolysis occurs along with the loss of antitumor activity (5, 6).

The maximum activity of these drugs is through the S-phase of the cell cycle and a prolonged exposure is required for optimal efficacy (6, 7). Therefore, oral administration is the main approach for achieving prolonged drug exposure as well as improving patient compliance and preferred over continuous intravenous administration. However, camptothecin derivatives show low oral bioavailability that may be explained by their physicochemical factors such as solubility and permeability in addition to physiological factors (*i.e*. intestinal absorption, cytochrome P_450_-dependent high first pass effect and efflux by a variety of transporters mostly P-glycoprotein) (8).

Similar to other lipophilic analogues of camptothecin, low and variable oral bioavailability of 9-NC was observed in pharmacokinetic studies in rats (9). It is currently unknown as to whether physiological factors play a role in contributing to the low and variable bioavailability of 9-NC.

Biodegradable nanoparticles have been suggested as being promising drug carriers with the goal of improving oral bioavailability by circumventing P-gp exposure thereby protecting labile molecules from gastrointestinal (GI) enzymes. In addition they provide prolonged drug exposure of target cells (for example cancerous cells) by providing sustained drug release in the circulation (10-14).

Consequently, the encapsulation of camptothecins within biocompatible nanocomposites such as liposomes, nanocapsules, micellar systems and conjugates has been proposed as a method to increase their oral bioavailability (14-17). The encapsulated drug is not expected to have an increased mean residence time within the body and it is not anticipated to be recognized by P-gp. Furthermore encapsulation can stabilize the labile lactone structure resulting from a low gastric pH. (18, 19)

As the evaluation of the intestinal absorption of the encapsulated 9-NC as compared to the free drug is of specific importance and there was no previous study carried out regarding this matter, a decision taken to investigate the uptake and transport characteristics of 9-NC polymeric nanoparticles in the human colon adenocarcinoma cell line, Caco-2 cell, as an *in-vitro *model for intestinal absorption.

Caco-2 cells are a human colon epithelial cancer cell line, commonly used as an *in-vitro *model to study the human intestinal absorption of drugs and new developed carriers particularly nanoparticles (20-26). When cultured as a monolayer, Caco-2 cells differentiate to form tight junctions between cells serving as a model of the paracellular movement of compounds across the monolayer. In addition, Caco-2 cells express transporter proteins, efflux proteins, Phase II conjugation enzyme and serve as a model for a variety of transcellular pathways in addition to the metabolic transformation of test substances (23-26).

## Experimental

9-Nitrocamptothecin (9-NC), 99.8% pure, was purchased from Yuanjian Pharmaceutical Technology Develop Co., (China). Poly (DL, lactide-co-glycolid) (PLGA, 50:50 MW 12000) was obtained from Boehringer Ingelheim Co. (Ingelheim, Germany) in the form of Resomer(R) 502H. Polyvinyl alcohol (PVA, MW 30000 Da, 87% hydrolyzed) was donated by Mowiol (Germany).

The Caco-2 cell line was obtained from the American Type Culture Collection (ATCC, Rockville, MD). Dulbecco,s modified eagle medium (DMEM), heated inactivity fetal bovin serum (FBS), non-essential aminoacids, *L*-glutamine, sodium pyruvate, trypsin-EDTA (0.025%), penicillin-streptomycin solution, Hanksۥ balanced salt solution (HBSS) and *N*-2-hydroxyethyl piperazine-*N*-2-ethanesulfonic acid (HEPES) were purchased from Gibco (Invitrogen, Carlsbad, CA). 6-coumarine was purchased from (Fisher science, USA).

Acetone (Acet), pure potassium dihydrogen phosphate, Dichloromethane (DCM) and acetonitril were of HPLC or pharmaceutical grade (Merck, Germany).

*Preparation of various diameter nanoparticles*

Nanoparticles of diameters 110, 190, 310, 520 and 980 nm were formulated by the nanoprecipitation method and were characterized as discribed in our previous report (27). Briefly, the 9-NC (1 mg) and PLGA polymer were dissolved in the organic phase. For smaller nanoparticles (110, 190 nm) acetone was used as solvent. The solvent of choice used for the particles of sizes 310, 520 and 980 nm was DCM. The amount of polymer utilized depended on the desired size of the particles (Table 1). The organic phase was added drop wise 0.5 mL/min into a PVA aqueous solution (pH was adjusted to 3 by 0.1 N HCl) and stirred magnetically (700 rpm) at room temperature until complete evaporation of the organic solvent was achieved.

Subsequently, the prepared nanoparticles were accumulated by ultracentrifugation (Beckman, XL-90) at 45000 rpm and 4°C for 1 h. To obtain nanoparticles of 110 nm in diameter, the nanoparticle suspension (N1) was filtered through 0.2 μm syringe filters prior to ultracentrifugation.

**Table 1:** Formulations and characterization parameters of 9-NC nanoparticles (n = 3).

**Loading (%)**	**PI**	**Size** **(nm)**	**Aqueous phase (mL)**	**Organic phase (mL)**	**PVA (%)**	**PLGA (mg)**	**Sample**
30.5 ± 2.87	0.22	110 ± 8.51	30	Acet, 15	1	50	**N1**
35.5 ± 4.15	0.28	190 ±12.67	20	Acet, 12	1.10	165	**N2**
42.5 ± 3.20	0.26	310 ± 13.35	40	DCM, 20	1.50	50	**N3**
40.5 ± 4.51	0.25	523 ± 10.67	40	DCM, 20	1.50	100	**N4**
47.6 ± 3.14	0.21	950 ± 14.53	25	DCM, 10	1	100	**N5**

PI: Polydispersity Index

The nanoparticles were washed three times in distilled water (pH 3) to allow for the complete removal of free drug and excess surfactants then freeze-dried. The acidic conditions of this procedure stabilized the active lactone form of the drug used.

The size distribution of the nanoparticles and their overall size was measured by laser light scattering (Brookhaven Instruments, Worcestershire, UK). The size distribution was achieved by using the polydispersity index. The lower the value is, the narrower the size distribution or the more uniform the nanoparticle sample is. The data reported in Table 1 represents an average of five recorded measurements.

The morphology and surface characteristics of the nanoparticles were tested using scanning electron microscopy (SEM) (Phillips, Eindhoven, Netherlands.) (27).

The drug content within the PLGA nanoparticles was determined as follows. 10 milligrams of freeze dried nanoparticles were dissolved in 1 mL DCM. The organic solvent was evaporated under a gentle stream of nitrogen. The residue was then dissolved in 1 mL of mobile phase and the concentration of 9-NC was analyzed by the previously reported HPLC method (28).

*In-vitro drug release*

The *in-vitro *drug release of the nanoparticles was determined after suspending 10 milligrams of the 9-NC nanoparticles in 50 ml of phosphate buffer solution (pH 7.4, 37°C) then placing in a shaking water bath (200 rpm). At designated time intervals, 0.5 mL of sample was removed and centrifuged at 15,000 rpm for 20 min. The supernatant (100 μL) was injected directly into the HPLC instrument and the amount of released drug was determined. The precipitated nanoparticles were then redispersed in fresh 0.5 mL release medium and placed back into the original release medium for continuous measurement.

*Caco-2 cell culture*

Caco-2 cells were grown as a monolayer in 150 cm^2^ plastic culture flasks in Dulbecco’s modified eagle medium (DMEM) supplemented with 10% (v/v) fetal bovine serum (FBS), 1% (v/v) non-essential amino acid solution, Na-Pyruvate and penicillin-streptomycin at 37°C in an atmosphere consisting of 5% CO2 and 90% relative humidity. Cells were passed 1: 4, every 5 days (at 70-80% confluence) using trypsine-0.025% EDTA. To study the transport and uptake passages 30-35 were used to estimate the GI barrier for oral chemotherapy.

*Transport study*

For the transport studies, cells (80% confluent) were harvested with trypsine–0.025% EDTA and seeded at a density of 10^5^ onto a polystyrene insert (1.131 cm^2^ growth area, Costar, Cambridge, MA) within the 12-well culture plates. The culture medium (0.5 mL in the apical side and 1.5 mL in basolateral side) was replaced 5 days following seeding and every 2 days thereafter.

The quality of the monolayers was assessed by measuring their transepithelial electrical resistance (TEER) at 37°C using an EVOM epithelial Voltmeter with an Endohm electrode (World Precision Instruments, INC., Sarasota, FL.) Also the transport of Lucifer yellow across the cell layer was determined at the end of each experiment. Only monolayers displaying TEER values above 400 Ω were used in the experiments. The permeability of Lucifer yellow was determined to be < 1 % in all of the conducted experiments.

The transport study across the Caco-2 cell monolayers was carried out using a monolayer 21 days post seeding. Before the experiments, the monolayers were washed with HBSS containing 0.01 M HEPES (pH 7.4) and the TEER was measured. Then monolayers were then pre-incubated at 37°C for 30 min and the TEER was subsequently measured again. The HBSS on both sides of the monolayer was removed via aspiration. For the transport study of the nanoparticles and free drug, 0.5 ml HBSS (pH 7.4) containing nanoparticles and drug solution was added on the apical side and 1.5 mL of HBSS (pH 7.4) was added on the basolateral side of the monolayers. Then 200 μL aliquots were removed from the receiver side at desired time intervals (0.5-3 h). The concentration of drug in the samples was determined using the HPLC method (28).

*Caco-2 cell uptake study*

Cells (80% confluent) were harvested with trypsine-0.025% EDTA solution and cultured in a 96-well black plate (Costar, Corning Incorporated) at a density of 5 x 10^5^ cells/well. When the cells within the 96-well plate reached almost 90% confluence the medium was removed. The cells were then washed with 200 μL HBSS and equilibrated for 1 h in an incubator. Upon the removal of the HBSS, appropriate amounts consisting of 200 μL of the 9-NC nanoparticles or a control equivalent to the predesigned concentration were introduced into each well. For each nanoparticle preparation and the control (no particles or drug solution) a total of 8 wells (one column) were used.

After 3 hours of incubation the nanoparticle suspension was removed by plastic pipette, the sample columns were washed three times with cold Phosphate buffer solution and the cells were solubilized in 50 μL solution of 0.5% triton-X 100 for 30 min.

Following the addition of 200 μL of the mobile phase, the samples were centrifuged for 10 min at 13,000 rpm and injected into the HPLC. In order to test the effect of incubation time and drug concentration on the nanoparticle uptake, cells were incubated with different concentrations of the drug for 3 h or the experiments were performed utilizing different incubation times.

*Confocal laser scanning microscopy*

Caco-2 cells were grown in Bioptech Delta T-dishes (Lab-Tek Chambered Coverglass system) and maintained with 5% CO_2_ at 37°C. After 80% confluence, the medium was removed and washed with HBSS. The cells were then incubated along with a suspension of 6-coumarine nanoparticles in the HBSS for 1-3 h.

In brief, for the preparation of coumarine-6 nanoparticles a solution of 0.25 mg coumarine in acetone (2 mL) was emulsified in 20 mL of an aqueous solution of 1% PVA using stirring at 700 rpm. Following the evaporation of the organic solvent, the nanoparticles were separated by an ultracentrifuge instrument at 40000 rpm and washed three times with distilled water. In order to remove the excess amount of coumarine, the nanoparticle suspension (10 mg in 50 mL water) was dialyzed through a dialysis membrane in 500 mL distilled water for 3 h.

At the end of the incubation periods, the cell monolayer was rinsed three times with cold PBS to remove excess nanoparticles and/or free dye. Subsequent to adding fresh HBSS buffer the cells were viewed and imaged under a confocal laser scanning microscope (Carl Zeiss LSM 410, Goettingen, Germany) using a FITC filter (Ex (λ) 540 nm, Em (λ) 560 nm). The images were then processed with the aid of Carl Zeiss LSM software.

*Statistical analysis*

A one-way ANOVA was performed to compare the uptake and transport parameters between the 9-NC nanoparticles and free drug groups. The level of significance was p < 0.05.

## Results

Camptothecin derivatives are potent anticancer drugs that show low oral bioavailability due to their low solubility, high first pass metabolism effect and effluxes via a variety of transporters mostly P-glycoprotein (8). Today encapsulation of these drugs within biodegradable nanoparticles is proposed as an alternative method for increasing their oral bioavailability (15-18).

Oral absorption of polymeric nano and microparticles by the gastrointestinal tract (GI) has been extensively studied for the last two decades (29-32). The possible mechanisms for transport of the particles through the gastrointestinal (and other physiological) barriers could be (1) paracellular passage: particles ‘‘kneading’’ between intestinal epithelial cells due to their extremely small size (< 50 nm); (2) endocytotic uptake of the particles: absorbed by intestinal enterocytes through endocytosis (particles size < 500 nm); and (3) lymphatic uptake: particles adsorbed by M cells of the Peyer’s patches (particle size < 5 mm) (11).

Cell culture models, such as Caco-2-cells offer the possibility of using pharmacological tools to generate more precise information on the mechanisms of uptake (20-26).

This model has been used to demonstrate the absorption characteristics of Poly acrylic acid (33), a conjugate of n-butyl cyanoacrylate (34), and PLGA nanoparticles (35, 36) by intestinal cells. However, reports are still conflicting with regards to the kinetic uptake in addition to the locations and mechanisms of uptake.

*Characterization of 9-NC PLGA nanoparticles*

The size of the nanoparticles ranged from 110 to 950 nm with a polydispersity of lower than 0.3 indicating a uniform particle size distribution (Table 1). The zeta potential of nanoparticles was about -25 mV. The drug loading was more than 30%, suggesting that these particles are suitable for cellular studies in addition to quantifying uptake and transport.

**Figure 2:**
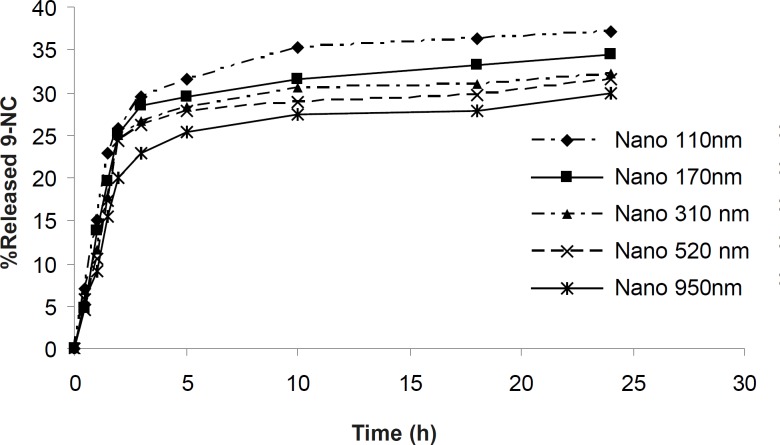
*In-vitro *release profile of 9-NC at PBS (pH 7.4) from PLGA nanoparticles

 Nanoparticles were found by SEM to be smooth and spherical in shape. The *in- vitro* drug release profiles of the 9-NC nanoparticles are shown in Figure 2. According to this data, at maximum 30% of the drug is released from nanoparticles during first 3 hours.

*Transepithelial transport across Caco-2 cell*

*monolayer*

**Figure 3:**
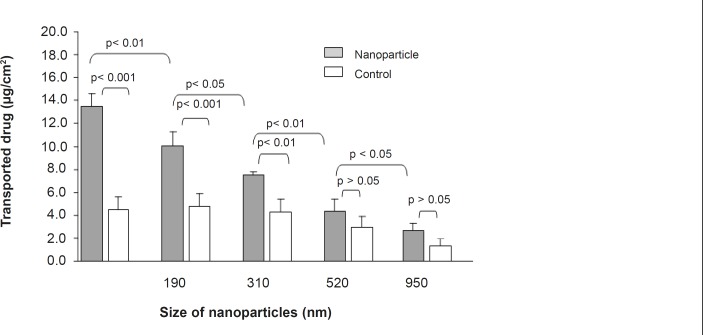
Effect of nanoaparticle diameter on 9-NC transport from apical to basolateral of Caco-2 cell monolayer. The control is 9-NC released under *in-vitro *conditions from various diameter nanoparticles and incubated with Caco-2 cells. (100 μg/mL, n = 6).

**Figure 4:**
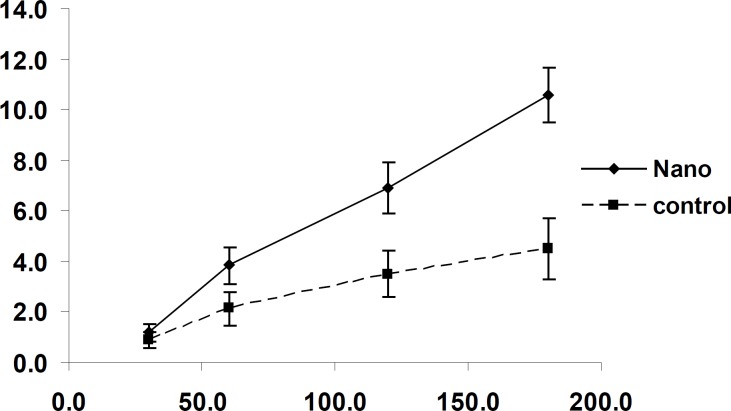
9-NC transport from the apical to basolateral side of the Caco-2 cell monolayer after 3 hours incubation of N_1_ formulation and control. The control is 9-NC released under *in-vitro *conditions from a nanoparticle formulation (N1) and incubated with Caco-2 cells. (100 μg/mL, n = 6).

The 9-NC apical to basolateral transport as either nanoparticle or drug solution was measured to evaluate the intestinal absorption (Figure 3 and 4). The drug encapsulated within smaller particles (110 nm) was more efficiently transported in comparison with the control and bigger particle sizes at the same concentration (Figure 3). For nanoparticles of size 110 nm the amount transported was roughly 3 times more than that of control and at each time point the permeated amount of 9-NC as nanoparticles exceeded that of the control (Figure 4). 

**Figure 5:**
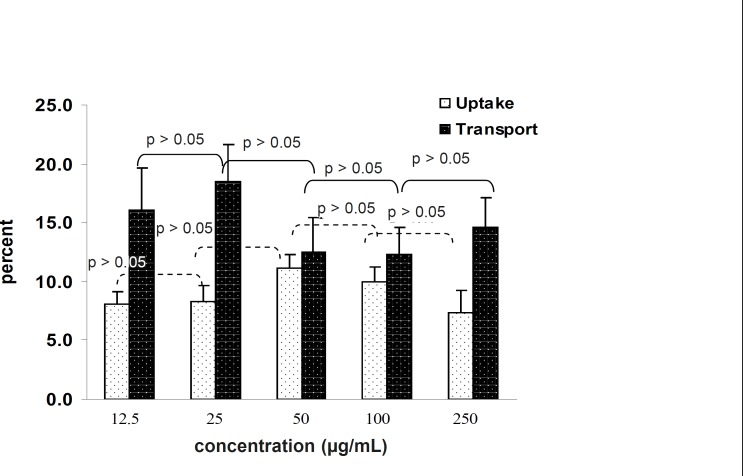
Effect of concentration on 9-NC uptake and transport from the apical to basolateral side of the Caco-2 cell monolayer after 3 h incubation with N_1_ formulation. (n = 6).

The amount of transported drug encapsulated inside the nanoparticles that passed through the Caco-2 cells increased by increasing the concentration in the incubation medium to a range of within 12.5 to 250 μg/mL (Figure 5). It is clear the percentage of transported drug is constant and there is no saturated pathway. 

**Figure 6:**
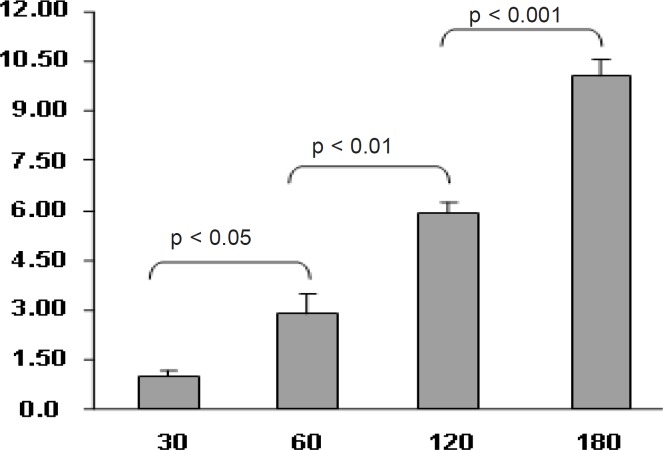
Effect of incubation time of nanoaprticles (N_1_ formulation) on 9-NC transport from the apical to the basolateral side of the Caco-2 cell monolayer. (100μg/mL, n = 6).

In another experiment, the effect of time on the transport of nanoparticles was studied. The amount of transported drug to basolateral side increased along with the incubation time up to 3 h. (Figure 6) 

In all of the studies, the control experiments were carried out by incubating Caco-2 cells with 9-NC released from nanoparticles in PBS (pH = 7.4) at 37°C over 3 h.

*Effect of time and concentration on uptake*

To determine the effect of time and concentration on the uptake of 9-NC by Caco-2 cells the experiment was carried out over different time intervals and concentrations. 

The tests carried out to messure the effect of concentration on cell uptake showed that the percentage of uptake is constant and does not follow a saturable pathway. In addition it was shown that increasing the concentration leads to an increase in uptake. (Figure 5) 

**Figure 7:**
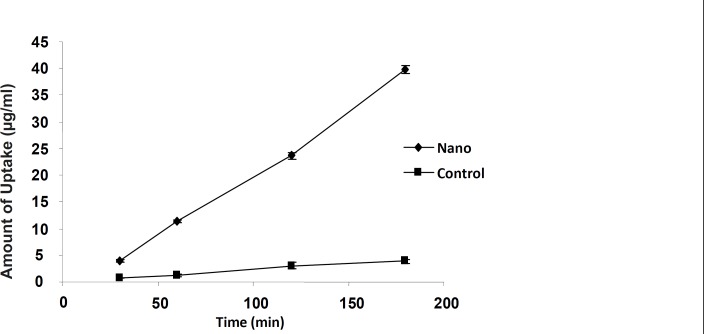
Effect of incubation time of nanoparticles (N_1_ formulation) on 9-NC uptake by Caco-2 cells. The control is 9-NC released under *in-vitro *conditions from a nanoparticle formulation (N1) and incubated with Caco-2 cells. (100 μg/mL, n = 6).

The uptake of nanoparticles by Caco-2 cells was time dependent and increased with time. (Figure 7) 

**Figure 8:**
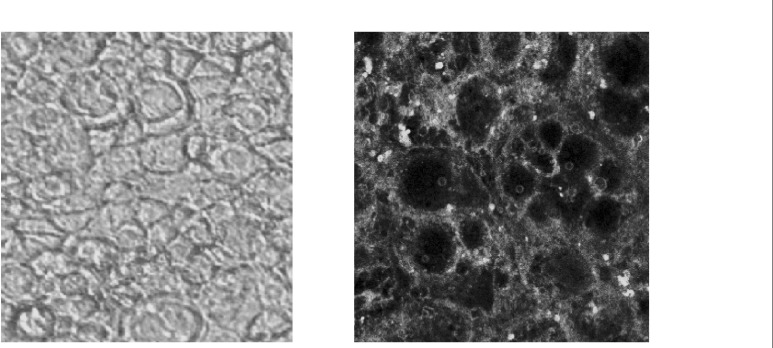
Confucian laser scanning microscopy: Interaction of 6-coumarine nanoparticles with the Caco-2 cell membrane (right), plain and intact Caco-2 cell (left).

Confocal microscopy of the cells exposed to 6-coumarine nanoparticles (size 130 nm) showed the nanoparticles were mostly localized in the cell membrane and could not enter within the cells. (Figure 8).

## Discussion

As the closed, active lactone ring is a structural requirement for the effective biologic activity of camptothecins, many researchers have investigated various modifications in an effort to promote lactone stability (37-41).

Discovery of lactone stabilization through lipid bilayer partitioning has led to the design of more lipophilic analogues in order to promote partitioning of these agents into the lipid bilayers of erythrocytes and protect the lactone from hydrolysis.

*In-vitro *and *in-vivo *preclinical studies suggest that the protracted administration of low doses of camptothecin analogues produces better antitumor activity than the less frequent administration of higher doses (41-43). Therefore, oral administration of 9-NC could mimic the protracted schedule and maximize patient convenience. However, the optimal oral dose and schedule are currently uncertain. In addition, oral administration of these analogues has been characterized by extensive inter and intra patient variability in bioavailability (41-43). At individual doses, there was a 4 to 16 fold variability in 9-NC exposure among different patients and there was no relationship between the dose and AUC_0-24h_ (41-43).

Recent studies showed that the encapsulation of these drugs in biodegradable nanoparticles was proposed as an alternative method to increase their oral bioavailability and lower variability in pharmacokinetic parameters (15-18). In a previous study, we evaluated the *in-vitro *cytotoxicity and *in-vivo *pharmacokinetic parameters of 9-NC loaded PLGA nanoparticles in rats. The results showed a significant improvement in the efficacy of the prepared carrier in the retention of 9-NC in both forms of the lactone and the total lactone and carboxylate form (44).

In the present study we investigated the potential of a new developed carrier for improving the intestinal transport of 9-NC by using a Caco-2 monolayer as an efficient *in-vitro *model.

The effect of incubation time, concentration and size of nanoparticles on 9-NC transport and uptake by Caco-2 cell was evaluated. For visualizing the interaction of the drug and the cell, coumarine nanoparticles were used. The cells were viewed and imaged under a confocal laser scanning microscope.

The results of the confocal microscopy, nonsaturable transport and uptake of nanoaparticles in various concentraions suggests that passive diffusion is predominant mechanism of uptake for this drug.

These results are in accordance with the Manisha and Labhasetwar data (36) obtained by studying the uptake of PLGA microparticles in various sizes (0.1, 1, 10 μm) with the Caco-2 cell monolayer. Their results demonstrated that the Caco-2 cell microparticle uptake significantly depends on the diameter of the carrier, as the uptake of 0.1 μm diameter nanoparticles was roughly 2.5 fold greater than that of the 1μm particles and 6 fold greater than the 10 μm diameter microparticles (36).

In a study on a novel chitosan nanoparticle, the confocal laser scanning microscopy observations confirmed that the nanoparticles were able to open the tight junctions between Caco-2 cells and allowed for the transport of nanoparticles via the paracellular pathways (36).

According to Win and Feng, particle surface coating in addition to particle size could affect the cellular uptake of nanoparticles (45). Their results illustrated that PLGA nanoparticles have a significantly higher level of cellular uptake compared with polystyrene nanoparticles. An observed plateau effect for the cellular uptake efficiency against the nanoparticle concentration or the cell incubation time suggested that the cellular uptake of the polymeric nanoparticle is saturable. The results demonstrated that nanoparticles of biodegradable polymers of small enough size and with appropriate surface coating may have great potential for being utilized as a means of orally delivering anticancer drugs as well as other therapeutic agents (45, 46).

In another study the cytotoxicity of doxorubicin, a P-gp substrate, incorporated into biodegradable polycyanoacrylate nanospheres was investigated in resistant cell lines (14). It was observed that cellular uptake was higher when doxorubicin was loaded into nanospheres. The cell uptake kinetics of doxorubicin nanoparticles was unchanged in the presence of cytochalasin B, an endocytosis inhibitor. Furthermore, efflux studies showed a similar profile for the drug for both the nanoparticulate and free forms. They suggested that nanoparticles did not enter the cell (14).

Our present investigation also showed that nanoparticles did not enter the cell, as such the higher transport of 9-NC using the nanoparticle formula may be explained by a local delivery of the drug in high concentration close to the cell membrane following the degradation of the polymeric carrier. Such a local microconcentration of 9-nitrocamptothecin was believed to be able to saturate P-gp, which in turn could overcome the P-gp mediated efflux of the drug.

Moreover, the increased stability of 9-nitrocamptothecin within the acidic microclimate of the PLGA polymeric matrix and therefore higher percentage of the lactone form of the drug that is more lipophilic than the related carboxylic form could result in increased diffusion and may well have facilitated the diffusion of 9-NC across the cell membrane (47). Prior data indicated a closed α-hydroxylactone ring is important for the passive diffusion of these drugs into cancer cells and for successful interaction with topo I (39-41).

The particles did not alter TEER over a 3 h period, demonstrating the lack of a paracellular component in the transport of PLGA particles. Confocal microscopy studies along with a lack of an effect on TEER support a transcellular uptake mechanism. However, more detailed mechanistic studies would need to be carried out to establish the uptake mechanism.

## Conclusion

To surmise, the transported amount of 9-NC loaded PLGA nanoparticles across Caco-2 monolayer was size dependent. The nanoparticles consisting of the smallest diameter (110 nm), showed significantly greater transport compared to those larger in size. The transport as well as the uptake of nanopartcles was independent of concentration. Transport increased by increasing the incubation time indicating the lack of a saturable transport process. The increased transport in the amount of 9-NC nanoparticles through the epithelial cells along with the sustained release characteristics of this labile drug indicate that PLGA nanoparticles may be considered as a promising carrier system for the oral administration of lipophilic anticancer drugs such as 9-nitrocamptothecin.

## References

[1] Garcia-Carbonero R, Supko GS (2002). Current perspectives on the clinical experience, pharmacology, and continued development of the camptothecins. Clinical Cancer Res.

[2] Gerrits CJH, Jonge MJA, Scgellens JHM, Stoter G, Verweij J (1997). Topoisomerase I inhibitors: the relevance of prolonged exposure for present clinical development. Br. J. Cancer.

[3] Iyer L, Ratain MJ (1998). Clinical pharmacology of camptothecins. Cancer Chemother. Pharmacol.

[4] Stehlin JS, Giovanella BC, Natelson EA, de Ipolyi PD, Coil D, Davis B, Wolk D, Wallace P, Trojacek A (1999). A study of 9-nitrocamptothecin (RFS-2000) in patients with advanced pancreatic cancer. Int. J. Oncol.

[5] Fassberg J, Stella VJ (1992). A kinetic and mechanistic study of the hydrolysis of camptothecin and some analogues. J. Pharm. Sci.

[6] Liu LF, Duann P, Ching-Tai L, D›Arpa P, Wu J (1996). Mechanism of action of camptothecins. Ann. NY Acad. Sci.

[7] Houghton PJ, Stewart CF, Zamboni WC, Thompson J, Luo X, Danks MK (1996). Schedule-dependent efficacy of camptothecins in models of human cancer. Ann. NY Acad. Sci.

[8] Pommier Y, Gupta M, Valenti M, Nieves-neira W (1999). Cellular resistance to camptothecins. Ann. NY Acad. Sci.

[9] Zhong DF, Li K, Xu JH, Du Y, Zhang YF (2003). Pharmacokinetics of 9-nitro-20(S)-camptothecin in rats. Acta Pharmacol. Sin.

[10] Brannon-Peppas L (1995). Recent advances on the use of biodegradable microparticles and nanoparticles in controlled drug delivery. Int. J. Pharm.

[11] Labhasetwar V (1997). Nanoparticles for drug delivery. Pharm. News.

[12] Moghimi SM, Hunter AC, Murray JC (2001). Long-circulating and target specific nanoparticles: theory and practice. Pharmcol. Rev.

[13] Panyam J, Labhasetwar V (2003). Biodegradable nanoparticles for drug and gene delivery to cells and tissue. Adv. Drug Deliv. Rev.

[14] Brigger I, Dubernet C, Couvreur P (2002). Nanoparticles in cancer therapy and diagnosis. Adv. Drug Deliv. Rev.

[15] Drummond DC, Meyer O, Hoong K, Kirpotin DB, Papahadjopoulos D (1999). Optimizing liposomes for delivery of chemotherapeutic agents to solid tumors. Pharmacol. Rev.

[16] Onishi H, Machida Y (2003). Antitomur properties of irinotecan-containing nanoparticles prepared using poly (DL-lactic acid) and poly (ethylen glycol)-block-poly (propylene glycol)-block-poly (ethylene glycol). Biol. Pharm. Bull.

[17] Zhang L, Hu Y, Jiang X, Yang C, Lu W, Yang YH (2004). Camptothecin derivative-loaded poly (caprolactone-co-lactide)-b–PEG-b-poly(caprolactone-co-lactide) nanoparticles and their distribution in mice. J. Control. Release.

[18] Chen H, Lamger R (1998). Oral particulate delivery: status and future trends. Adv. Drug Deliver. Rev.

[19] Ponchel G, Irache J-M (1998). Specific and non-specific bioadhesive particulate systems for oral delivery to the gastrointestinal tract. Adv. Drug Deliver. Rev.

[20] Gan LS, Thakker DR (1997). Application of the Caco-2 model in the design and development of orally active drugs: elucidation of biochemical and physical barriers posed by the intestinal epithelium. Adv. Drug Deliver. Rev.

[21] Artursson P (1990). Epithelial transport of drugs in cell culture. I: A model for studying the passive diffusion of drugs over intestinal absorptive (Caco-2) cells. J. Pharm. Sci.

[22] Bvan Breemen R, Li Y (2005). Caco-2 cell permeability assays to measure drug absorption. Expert Opinion on Drug Metabolism and Toxicology.

[23] Gaumet M, Gurny R, Delie F (2009). Localization and quantification of biodegradable particles in an intestinal cell model: the influence of particle size. Eur. J. Pharm. Sci.

[24] Kolhatkar RB, Swaan P, Ghandehari H (2008). Potential oral delivery of 7-ethyl-10-hydroxy-camptothecin (SN-38) using poly(amidoamine) dendrimers. Pharm. Res.

[25] Kitchens KM, Kolhatkar RB, Swaan PW, Eddington ND, Ghandehari H (2006). Transport of poly(amidoamine) dendrimers across Caco-2 cell monolayers: Influence of size, charge and fluorescent labeling. Pharm. Res.

[26] Moyes SM, Smyth SH, Shipman A, Long S, Morris JF, Carr KE (2007). Parameters influencing intestinal epithelial permeability and microparticle uptake in-vitro. Int. J. Pharm.

[27] Derakhshandeh K, Erfan M, Dadashzadeh S (2007). Encapsulation of 9-nitrocamptothecin, a novel anticancer drug, in biodegradable nanoparticles: factorial design, characterization and release kinetics. Eur. J. Pharm. Biopharm.

[28] Derakhshandeh K, Dadashzadeh S (2005). Liquid chromatography quantitation of the lactone and the total of lactone and carboxylate forms of 9-nitrocamptothecin in human plasma. J. Chromatogr. B.

[29] Eyles J, Alpar HO, Field WN, Lewis DA, Keswick M (1995). The transfer of polystyrene microspheres from the gastrointestinal tract to the circulation after oral administration in the rat. J. Pharm. Pharmacol.

[30] Florece AT, Hillery AM, Jani PU (1995). Factors affecting the oral uptake and translocation of polystyrene nanoparticles: histological and analytical evidence. J. Drug Target.

[31] Artrsson P, Palm K, Lutheman K (1996). Caco-2 monoayers in experimental and theoretical predictions of drug transport. Adv. Drug Deliv. Rev.

[32] Delie F, Rubas W (1997). A human colonic cell line sharing similarities with enterocytes as a model to examine oral absorption. Advatages and limitations of the Caco-2 model. Crit. Rev. Ther. Drug Carr. Syst.

[33] Kriwt B, Kissel T (1996). Poly (acrylic acid) microparticles widen the intercellular spaces of Caco-2 cell monolayer: an examination by confocal laser scanning microscopy. Eur. J. Pharm. Biopharm.

[34] Hillery AM, Toth I, Florence AT (1996). Co-polymerised peptide particles (CPP) I: synthesis, characterization and in-vitro studies on a novel oral nanoparticulate delivery system. J. Control. Rel.

[35] Mc Clean S, Prosser E, Meehan E, O›Malle D, Clarke N, Ramtoola Z (1998). Binding and uptake of biodegradable poly- dl- lactide micro- and nanoparticles in intestinal epithelia. Eur. J. Pharm. Sci.

[36] Manisha PD, Labhasetwar V, Walter E, Levy RJ, Amidon GL (1997). The mechanism of uptake of biodegradable microparticles in Caco-2 cells is size dependent. Pharm. Res.

[37] Slichenmyer WJ, Rowinsky EK, Donehower RC, Kaufmann SH (1993). The current status of camptothecin analogues as antitumor agents. J. Natl. Cancer Inst.

[38] Potmesil M (1994). Camptothecins: from bench research to hospital wards. Cancer Res.

[39] Hertzberg RP, Caranfa MJ, Holden KG (1989). Modification of the hydroxy lactone ring of camptothecin: inhibition of mammalian topoisomerase I and biological activity. J. Med. Chem.

[40] Wani MC, Nicholas AW, Manikumar G, Wall ME (1987). Manikumar G and Wall ME. Plant antitumor agents. 25. Total synthesis and antileukemic activity of ring A substituted camptothecin analogues. Structure-activity correlations. J. Med. Chem.

[41] Zamboni WC, Bowman LC, Tan M (1999). Interpatient variability in bioavailability of the intravenous formulation of topotecan given orally to children with recurrent solid tumors. Cancer Chemother. Pharmacol.

[42] Gupta E, Luo F, Lallo A, Ramanathan S, Vyas V, Rubin E (2000). The intestinal absorption of camptothecin, a highly lipophilic drug, across Caco-2 cells is mediated by active transporter(s). Anticancer Res.

[43] Schoemaker NE, Mathot RAA, Schoffski P, Rosing H, Schellens JHM, Beijnen JH (2002). Development of an optimal pharmacokinetic sampling schedule for rubitecan administered orally in a daily times five schedule. Cancer Chemother. Pharmacol.

[44] Dadashzadeh S, Derakhshandeh K, Shirazi FH (2008). 9-nitrocamptothecin polymeric nanoparticles: cytotoxicity and pharmacokinetic studies of lactone and total forms of drug in rats. Anticancer Drugs.

[45] Win KY, Feng S-S (2005). Effects of particle size and surface coating on cellular uptake of polymeric nanoparticles for oral delivery of anticancer drugs. Biomaterials.

[46] Orafai H, Kallinteri P, Garnett M, Huggins S, Hutcheon G, Pourcain C (2008). Novel poly (glycerol-adipate) polymers used for nanoparticle making: a study of surface free energy. Iranian J. Pharm. Res.

[47] Shenderova A, Bruke TG, Schewendeman SP (1999). The acidic microclimate in poly (lactide-co-glycolide) microsopheres stabilizes camptothecins. Pharm. Res.

